# Oscillatory Hypoxia Can Induce Senescence of Adipose-Derived Mesenchymal Stromal Cells Potentiating Invasive Transformation of Breast Epithelial Cells

**DOI:** 10.3390/cancers16050969

**Published:** 2024-02-28

**Authors:** Ashkan Novin, Khadija Wali, Aditya Pant, Shaofei Liu, Wenqiang Du, Yamin Liu, Lichao Wang, Ming Xu, Binsheng Wang, Yasir Suhail, Kshitiz

**Affiliations:** 1 Department of Biomedical Engineering, University of Connecticut, Storrs, CT 06269, USA; novin@uconn.edu (A.N.); wali@uchc.edu (K.W.); pant_aditya@yahoo.com (A.P.); shaofei.liu_sr@uconn.edu (S.L.); yasir.suhail@uconn.edu (Y.S.); 2 Department of Biomedical Engineering, University of Connecticut Health, Farmington, CT 06032, USA; wdu@uchc.edu (W.D.); yamin.liu@uconn.edu (Y.L.); 3 Department of Immunology, University of Connecticut Health, Farmington, CT 06032, USA; licwang@uchc.edu (L.W.); mixu@uchc.edu (M.X.); 4 Center for Aging Research, University of Connecticut Health, Farmington, CT 06032, USA; binwang@uchc.edu; 5 NEAG Comprehensive Cancer Center, University of Connecticut Health, Farmington, CT 06032, USA

**Keywords:** oscillatory hypoxia, fluctuating hypoxia, adipocyte-derived stromal cells, breast cancer, breast cancer dissemination, stromal invasion, stromal dissemination, breast epithelial transformation, MCF10A, MCF10A1, ASC, MSC recruitment, tumor microenvironment

## Abstract

**Simple Summary:**

Obesity is associated with increased occurrence and metastasis of breast cancer. Breast cancers are also very hypoxic and rich in adipose (fat) tissue. We found that adipocyte-derived stromal cells (ASCs) can transform normal breast epithelial cells to become invasive, potentiating them toward a cancerous state. We also found that unstable or fluctuating hypoxia, which is common in the tumor environment, can cause ASCs to become senescent, with an additive effect on breast epithelial potentiation to an invasive state.

**Abstract:**

Obesity is strongly associated with occurrence, metastasis, and resistance to therapy in breast cancers, which also exhibit high adipose content in the tumor microenvironment. Adipose tissue-derived mesenchymal stromal cells (ASCs) are recruited to breast cancer by many mechanisms, including hypoxia, and contribute to metastatic transition of the cancer. Breast cancers are characterized by regions of hypoxia, which can be temporally unstable owing to a mismatch between oxygen supply and consumption. Using a high-sensitivity nanopatterned stromal invasion assay, we found that ASCs could promote stromal invasion of not only breast cancer cell lines but also MCF10A1, a cell line derived from untransformed breast epithelium. RNA sequencing of MCF10A1 cells conditioned with medium from ASCs revealed upregulation of genes associated with increased cell migration, chemotaxis, and metastasis. Furthermore, we found that fluctuating or oscillating hypoxia could induce senescence in ASCs, which could result in an increased invasive potential in the treated MCF10A1 cells. These findings highlight the complex interplay within the breast cancer microenvironment, hypoxia, and the role of ASCs in transforming even non-cancerous breast epithelium toward an invasive phenotype, providing insights into early metastatic events.

## 1. Introduction

Progression of cancer is now widely considered to be an integrated outcome of both the genetic state of transformed cells as well as the tumor microenvironment (TME) [1]. The stromal component of the TME has been shown to promote cancer growth, TME inflammation, as well as the transition of cancer into a metastatic disease [2]. In breast cancers, stromal cells have been found to play a critical role at nearly every stage of the metastatic cascade [3]. Harnessing the large differences in mammalian rates of cancer metastasis, we have shown that stromal control of carcinoma invasion is both a selected and a regulated phenotype [4,5], providing an evolutionary basis for the role of stromal cells’ complicity in the invasive characteristics of cancer.

Breast cancer occurrence, as well as the rate of metastasis, are strongly correlated with obesity, suggesting that fat tissue may have a role to play in the pathogenesis of breast cancers [6,7]. Both luminal type A breast cancer, which is more common in occurrence, as well as the basal subtypes correlate strongly with obesity [8]. Among other cell types, breast stroma is highly enriched in adipocytes, a rich source of adipocyte-derived stromal cells (ASCs). ASCs are known to be recruited to the tumor site, particularly toward the hypoxic region [9,10]. It has been shown that recruited mesenchymal stromal cells in hypoxic breast cancer can promote the transition to a metastatic state by paracrine crosstalk in the TME, facilitated by the activation of Hypoxia Inducible Factor-1 (HIF-1) in hypoxia [11,12,13].

In recent years, it has been shown that the oxygen levels in hypoxic tumor cores are not stable and can fluctuate [14,15,16,17]. Many potential causes can contribute to this instability in O_2_ tension, including pulsation in poorly formed tumor vasculature, mismatch between O_2_ consumption and supply, temperate fluctuations, as well as heterogeneity in the metabolic state of cells [18,19,20,21]. We have also previously shown that highly glycolytic and hypoxic tumor cores may exhibit fluctuations in the transcriptional response of HIF-1, further contributing to the heterogeneity in the hypoxic TME [22]. Our own work, as well as that of others, suggest that fluctuations in hypoxia can result in a very different transcriptional and phenotypic response in cancers, resulting in gene signatures associated with various cancer types and adverse survival rates in breast cancer patients [23]. However, the effect of oscillatory hypoxia on other stromal cells has not been yet documented. Here, we used a nanoengineered stromal invasion assay to surprisingly discover that the presence of ASCs in the stromal compartment not only increases the invasive capacity of breast cancer cell lines MCF7A and MDA-MB-231 but also of the non-transformed breast epithelial cell line MCF10A1. Furthermore, we found that fluctuating hypoxia can induce senescence in ASCs. We showed that the presence of senescent ASCs can further enhance MCF10A1 migration, as well as their invasive capacity, suggesting the importance of cancer–stroma–TME crosstalk and its regulation in hypoxia as a mediator of breast cancer dissemination and metastasis.

## 2. Methods

### 2.1. Cell Sourcing and Maintenance

Fat-derived ASCs (preadipocytes) were isolated from healthy lean kidney donors aged 39 ± 3.3 y with a body mass index of 26.6 ± 0.9 (mean ± SEM) [24]. BJ, MDA-MB-231, and MCF7 cells were purchased from the American Type Culture Collection (ATCC), while MCF10A cells were purchased under an MTA from the Karmanos Cancer Institute.

MDA-MB-231 and MCF7 cells were cultured in DMEM supplemented with 10% FBS, 2 mM L-glutamine, and 1% penicillin–streptomycin. MCF10A1 cells were maintained in DMEM/F-12 medium supplemented with 5% horse serum, 20 ng/mL epidermal growth factor (EGF), 10 ug/mL insulin, 0.5 mg/mL hydrocortisone, and 10 mM HEPES. ASCs were also cultured in a-MEM supplemented with 10% FBS, L-glutamine, and 1% penicillin–streptomycin. BJ cells were purchased from the ATCC and were maintained in Cascade Human Fibroblast Expansion Basal Medium (Medium 106, Gibco, ThermoFisher Scientific Inc., Carlsbad, CA, USA) with 10% FBS, 1% ITS supplement (insulin, transferrin-sodium selenite) (Gibco, ThermoFisher Scientific Inc., USA), and 1% penicillin–streptomycin. BJ cells were always maintained in <75% confluency to prevent transformation, except in ANSIA, where completely confluent monolayers were warranted.

### 2.2. RNA Sequencing and Bioinformatics

RNA was isolated from MDA-MB-231 cell lysates after co-culture and sequenced with paired-end libraries on the Illumina platform. The RNA reads were aligned to the GRCh38 human genome with hisat2 [25]. The aligned reads were counted against the gene models using feature counts [26]. The read counts were analyzed with DESeq2 [27] on the R platform for differential expression. Custom scripts comparing the means of the differential expression z scores computed from the log2fold changes and GSEA [28] were used for the pathway analysis for different figures. Heatmaps were drawn after first transforming the read counts into transcript per million (TPM) values and then being normalized gene-wise.

The breast cancer single-cell RNA sequencing count matrix downloaded from GSE176078 was normalized and scaled. A total of 14 PCA components were used to run the non-linear dimensional reduction with a resolution of 0.8 (calculated by Seurat 4.0.52). MSC clusters were identified by the mesenchymal markers (PDGFRB and VIM) and stem cell markers (ALDH1A1, KLF4, LEPR). For each of the three breast tumor subtypes, HER2, TNBC and ER, from which MSC cells were identified, the mean scaled gene expression value (Z score) was calculated for the senescence, SASP, and oscillatory hypoxia gene sets. The *p*-values for the correlation of the z-scores were calculated using Pearson’s method.

### 2.3. Invasion Assay

To create patterned co-cultures, invasive cells (5 × 10^5^) were pre-labeled with CellTracker Green and seeded onto the culture plate. Following overnight incubation to ensure cell attachment, the stencil was carefully removed using blunt-end tweezers, revealing a defined cell-free area. Unlabeled stromal cells (5 × 10^5^) were then seeded to fill the vacated area previously covered by the stencil. After a 5 h incubation period allowing for stromal cell attachment, the culture plate was gently washed to remove any unattached cells. Finally, the plate was mounted onto the live microscopy stage for subsequent imaging and analysis.

### 2.4. Invasion Characteristics Quantification

Imaging was performed using a Zeiss Observer Z1 microscope equipped with an epifluorescence and phase-contrast setup within a tri-gas incubator. A multi-location scanning stage with 130 × 100 steps and Definite Focus v2 technology facilitated the automated image acquisition. Cells were imaged using a 10X/0.8 WD-0.55 EC Plan-Apochromat objective at 1 h intervals for a total duration of 24 h.

Invading cancer cells were monitored for 24 h using live epifluorescence microscopy, capturing images at 1 h intervals. The invasion fronts were tracked and quantified using the Region of Interest (ROI) panel in Fiji software. The underlying anisotropic nanotextured substrate induced highly directional invasive protrusions, amplifying the invasion process while enabling efficient data analysis due to the reduced dimensionality. The ROI selection involved manually tracing the invasion boundaries. The normalized extent of the invasion was calculated by dividing the total change in area by the initial length of the cancer–stroma interface.

The ROIs were then converted into one-dimensional profiles of the invasive front, as smoothed by a 20-pixel moving average filter. At each point along the profile, the mean signal intensity on either side (40 pixels each) was compared to the smoothed profile value. Peaks were identified based on the smoothed profile exceeding both adjacent side averages. Consecutive peaks were then identified by taking the midpoints of their respective intervals.

### 2.5. SA-βgal Staining of Cultured Cells

The SA-βgal activity of ASCs was detected by a histochemical process. In brief, cells were grown on a gas-permeable well plate, and after conditioning in Normoxia, Hypoxia, and Osc. Hypoxia, the cells were fixed and stained with SA-βgal detection solution. The samples were incubated at 37 °C without CO_2_ and protected from light overnight. The activity was determined by the detection of blue-stained cells under phase contrast.

### 2.6. Migration Assay Quantifications

Cancer cells were monitored for 12 h using live microscopy at 10 min intervals. The TrackMate plugin in Fiji was used to track the individual cells and the velocity, displacement, and directionality measurements.

## 3. Results

### 3.1. Adipocyte-Derived Stromal Cells (ASCs) Transform Breast Epithelial MCF10A1 to Assume Invasive Phenotype

**Figure 1:**
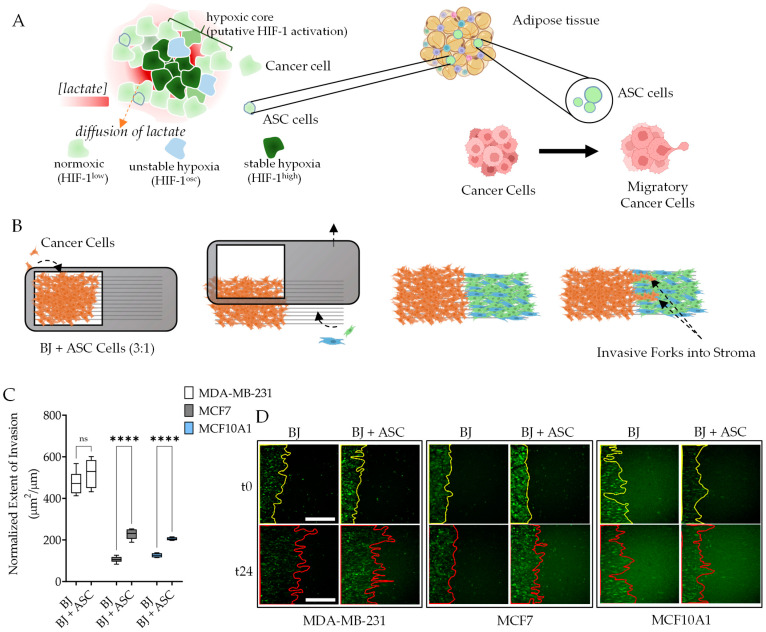
Adipocyte-derived stromal cells (ASCs) increase the invasiveness of breast cancer and normal breast epithelial cells. (**A**) (Left) Schematic of a tumor microenvironment (TME) containing breast cancer cells with a hypoxic core, which could also be unstable, as well as diffused metabolites (e.g., lactate). (Right) Adipose tissue can provide ASCs, which are recruited to the hypoxic tumor core and contribute to cancer metastasis. (**B**) Schematic of the ANSIA-based stromal invasion assay with ASCs present in the stromal compartment. (**C**) Normalized extent of the invasion of cancer cells (MDA-MB-231, MCF7, and MCF10A1) into the stromal compartment (containing BJ fibroblasts + ASC); (**D**) representative images at the first time point and the final time point (24 h) shown in the panel, with invasive fronts of breast epithelial cells (green) marked in yellow (0 h) or red (24 h) solid lines; scale bar is 500 microns. In (**C**), error bar: s.e.m.; ****: *p* < 0.0001; n.s. non-significant; n = 6 independent locations in ANSIA.

Obesity is correlated not only with breast cancer metastasis but also with tumorigenesis, particularly in post-menopausal women [29,30]. As fat tissue is a rich source of mesenchymal cells, which are known to be recruited to the site of breast cancers, we asked if ASCs could directly influence breast epithelial invasion into the surrounding connective stroma (Figure 1A). To test this hypothesis, we used MDA-MB-231, MCF7A, and MCF10A1 cells and measured their capability to invade stroma when ASCs were also present in the stromal compartment. MDA-MB-231 is representative of the highly aggressive triple-negative breast cancer (TNBC), and MCF7A represents the adherent derivation of a relatively less aggressive luminal type A cancer with estrogen receptor (ER)-positive and progesterone receptor (PR)-positive characteristics [31]. MCF10A1 represents the adherent mammary glandular epithelial cells, which are untransformed.

Stromal invasion by epithelia is a composite phenotype, consisting of not only the collective migration of epithelia but also likely many other co-occurring phenomena, e.g., disruption of matrix architecture and its remodeling, disrupting stromal cell–cell interaction, regulation of contractile force in stromal fibroblasts as well as epithelial cells, change in cell–cell interactions, as well as possibly chemotactic events. Therefore, studying stromal invasion in a physiological setting necessitates the presence of the matrix architecture, as well as a fibroblast-containing distinct space, where invasion could be measured. Typically, the epithelial invasion subsequent to the disruption of basal lamina activates fibroblasts, which are recruited to the site of injury in large numbers to stem the laminal injury. Based on these physiological considerations, we have previously described the Accelerated Nanopatterned Stromal Invasion Assay (ANSIA), a platform to measure stromal invasion by epithelial cells with high sensitivity [32]. The platform consists of monolayers of fluorescently labeled MCF10A1 cells and stromal fibroblasts forming a straight interface. Here, we used BJ fibroblasts only or those supplemented with ASCs at a 1:3 ratio to model the presence of recruited ASCs in the stroma (Figure 1B). The monolayers are patterned on an anisotropic array of nanogrooves, orthogonal to the interface between the epithelia-stroma monolayers. Using directional arrays of nanotextured fibers providing tactic cues for the overlaying monolayers, ANSIA reduces the movement of epithelial monolayer into stroma into a quasi-1-dimensional problem, while also accelerating the process of invasion, increasing the sensitivity of detection such that we can start detecting differences in conditions within 12 h of live observation [33].

Fluorescently labeled MDA-MB-231, MCF7A, and MCF10A1 cells were seeded on the ANSIA epithelial compartment. BJ only or the BJ+ASC mixture were patterned in the stromal region, and microscopy was performed after both cells had attached and formed complete monolayers (Figure 1B). In an observation period of ~24 h, we found that the presence of ASCs significantly increased the invasion of MCF10A1 as well as MCF7A cells. For MDA-MB-231, the presence of ASCs in the stroma did not result in a further increase in invasiveness, possibly because the basal invasiveness was already very high for MDA-MB-231 (Figure 1C,D). However, the dramatic effect of the presence of ASCs in the stroma on untransformed MCF10A1 cell invasiveness (~2-fold increase) suggests that recruitment of mesenchymal cells, even without tumor-generated hypoxia, may contribute to tumorigenesis and epithelial transformation. We, therefore, sought to question the effect of ASCs on MCF10A1 gene expression.

### 3.2. ASCs Enhance Invasion-Associated Gene Expression in MCF10A1 Breast Epithelial Cells

**Figure 2:**
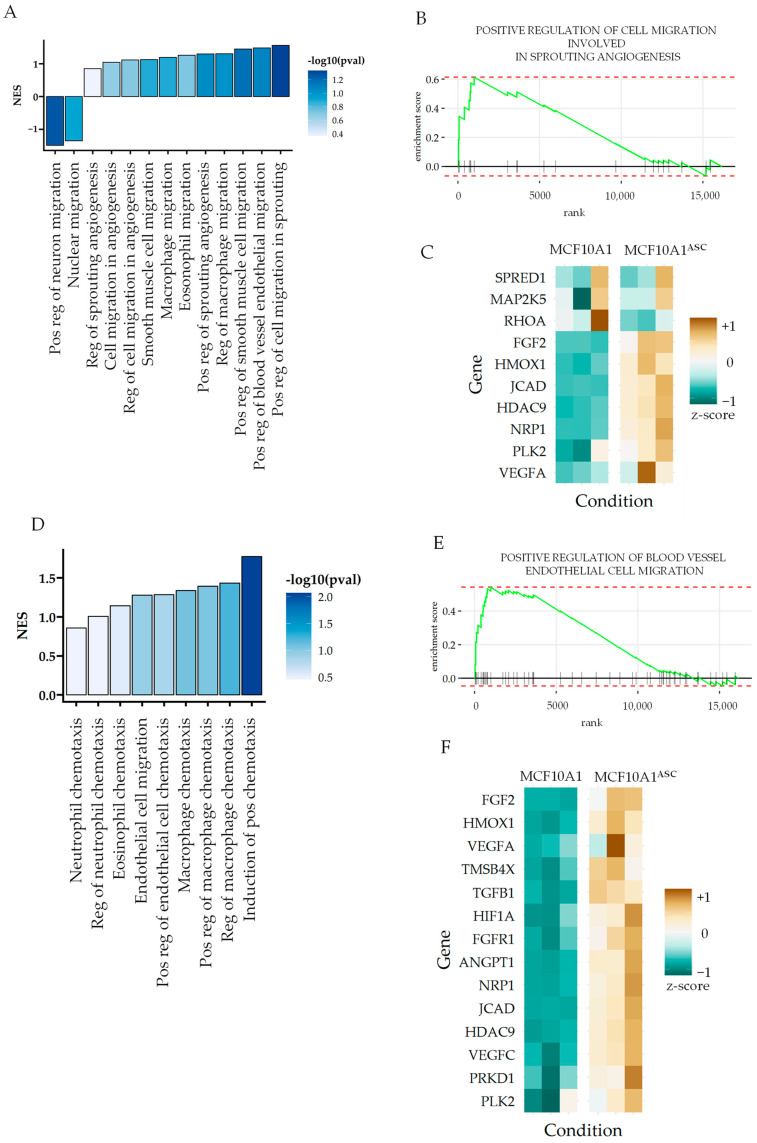
ASCs increase gene expression related to invasion in MCF10A1 cells. (**A**) Mean normalized enrichment score for selected gene ontologies (GOs) related to cell migration in MCF10A1 cells based on the differential gene expression (DFE) analysis after treatment with ASC medium. (**B**) Gene set enrichment analysis (GSEA) score ranked based on the positive regulation of cell migration involved in sprouting angiogenesis. (**C**) Relative expression of leading-edge genes for MCF10A1 and MCF10A1^ASC^. (**D**) Mean normalized enrichment score for selected GOs related to chemotaxis activated in MCF10A1 after ASC conditioning. (**E**) GSEA analysis for “Positive regulation of blood vessel endothelial cell migration” based on the DFE between MCF10A1 and MCF10A1^ASC^. (**F**) Relative expression of leading-edge genes.

To understand the paracrine effect of ASCs on breast epithelial cells, we treated MCF10A1 cells with conditioned medium from ASCs, or ASC medium components (control), for 48 h and performed RNA sequencing. Differential gene expression analysis revealed that treatment with ASC-conditioned medium resulted in significant upregulation of gene sets associated with cell migration and chemotaxis (Figure 2A). To identify the genes most contributing to the activation of these pathways, we performed gene set enrichment analysis (GSEA) for these ontologies. GSEA for a selected ontology “regulation of cell migration in sprouting angiogenesis” showed high enrichment, revealing leading-edge genes generally known to be key regulators of cell migration (Figure 2B). These included regulators of the RAS MAPK pathway, including SPRED1-encoding Spred-1 and MAP2K5, both of which are known to reduce tumor growth, although the latter promotes invasiveness in breast cancer [34,35] (Figure 2C). Similarly, the other endothelial cell migration pathway revealed other key genes, suggesting that ASCs were contributing to the transformation of MCF10A1 cells into a metastatic state (Figure 2D,E). These included isoforms of Vascular Endothelial Growth Factor, VEGFA and VEGFC, Hypoxia Inducible Factor 1 Alpha-Encoding HIF1A, Transforming Growth Factor Beta 1-Encoding TGFB1, and FGFR1-Encoding FGF Receptor 1 (Figure 2E,F). All these are key genes associated with the mesenchymal transition of epithelial cells, and they play a key role in regulating the metastasis of epithelial cancers. While supporting the increased invasiveness of MCF10A1 cells after conditioning with ASCs, the gene expression analysis suggests that ASCs may be contributing to the transformation of MCF10A1 epithelial cells into a more cancerous state.

### 3.3. ASCs Decrease Gene Expression Related to Cortical Actin and Epithelial Cell–Cell Adhesion

**Figure 3:**
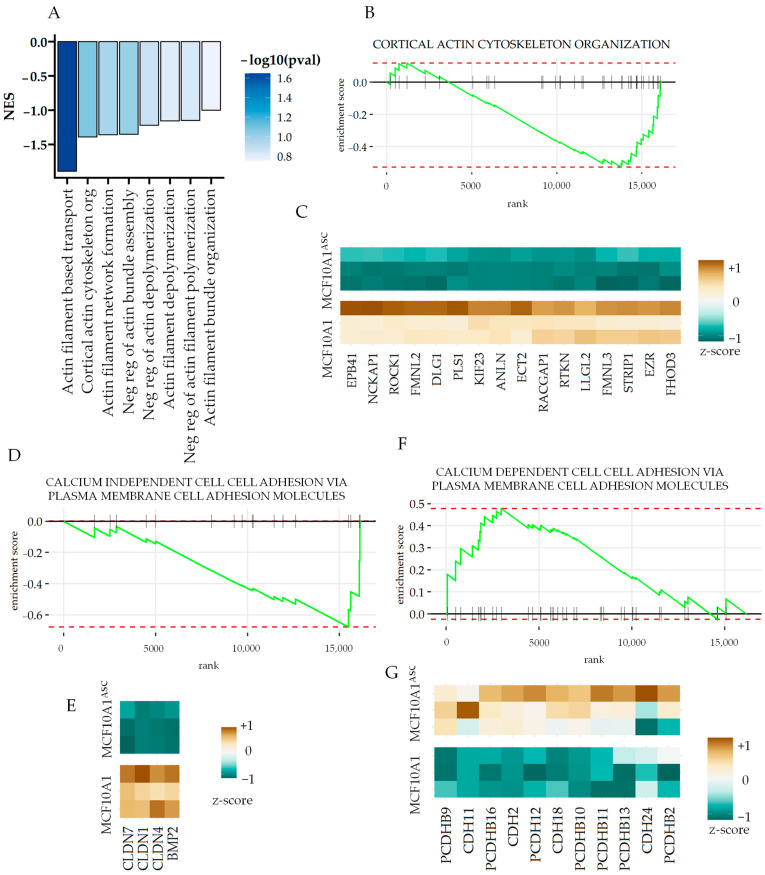
ASCs decrease gene expression related to cortical actin cytoskeleton and epithelial cell–cell adhesion. (**A**) Mean normalized enrichment score for selected gene ontologies (GOs) related to actin cytoskeletal organization in MCF10A1 cells based on the differential gene expression (DFE) analysis after treatment with ASC medium. (**B**) Gene set enrichment analysis (GSEA) for “Cortical actin organization”. (**C**) Relative expression of leading-edge genes for MCF10A1 and MCF10A1^ASC^. (**D**) GSEA analysis of genes changing between MCF10A1 and MCF10A1^ASC^ for GO: “calcium-dependent cell-cell adhesion molecules”, and (**E**) expression of leading edge genes. (**F**) GSEA analysis for “calcium-independent cell-cell adhesion molecules”, and (**G**) expression of leading-edge genes. In (**C**,**E**,**G**), the z-score for gene expression is shown.

While conditioned medium from ASCs increased the MCF10A1 cells’ migration and invasive properties, we also found that many pathways associated with actin polymerization were negatively enriched. Cortical actin cytoskeletal organization, actin filament bundle organization, as well as transport based on actin filament were negatively enriched (Figure 3A). GSEA analysis of “cortical actin cytoskeleton” revealed many key genes as leading edges, which provided a partial explanation for the increased invasiveness of MCF10A1 after ASC stimulation (Figure 3B,C).

Furthermore, ASC conditioning showed a negative enrichment of calcium-independent cell–cell adhesion (Figure 3D), revealing many claudins that decreased in expression in MCF10A1 cells (Figure 3E). Claudins are cell–cell adhesion desmosomes in tight junctions, which are critical for maintaining the epithelial layer integrity. A decrease in their expression is associated with a reduction in the epithelial phenotype and associated with carcinoma dissemination. In contrast, ASC conditioning showed enrichment in calcium-dependent cell–cell adhesion, revealing many genes encoding Cadherins and proto-cadherins (Figure 3F,G). However, the chief cadherin which increased in expression was CDH2, encoding N-Cadherin associated with mesenchymal transition of the breast epithelium. Cadherin switching from E-Cadherin to N-Cadherin (encoded by CDH1 and CDH2 respectively) is a strong prognostic marker of the invasive transformation of the breast epithelium. Another mesenchymal cadherin-encoding gene, CDH11, was also increased by ASC conditioning [36]. Cadherin-11 is necessary for metastatic spread in breast and pancreatic cancers [37], and it is remarkable that both CDH2 and CDH11 were increased in normal epithelial cells. Indeed, a dual N-Cadherin and Cadherin-11 targeting antibody has been shown to reduce breast cancer metastasis [37]. Interestingly, many clustered protocadherin-encoding genes were also upregulated by ASC conditioning, including PCDHB2,9,10,11,13, as well as PCDH12. Although little is known about their role in cancers, methylation of several clustered proto-cadherins has been found in cancers [38].

### 3.4. Oscillatory Hypoxia Promotes Senescence in ASCs

Stromal mesenchymal cells are recruited at the site of hypoxic breast cancers. However, recent reports have suggested that hypoxia in tumors can be unstable owing to the temporal dynamic equilibrium between oxygen supply and consumption. The tumor neovasculature is often leaky [39,40], and owing to the large number of packed cells, even a shift in glycolysis may still result in high consumption of O_2_. Furthermore, lactate in the environment can result in fluctuations in HIF-1 transcriptional activity [22]. We therefore tested how ASCs may be phenotypically different in oscillatory hypoxia.

We have recently identified genes that change in breast cancer MDA-MB-231 cells in response to oscillatory vs. stable hypoxia. These oscillatory-specific genes are strictly defined as not only those which differ significantly from stable hypoxia but are indeed regulated oppositely, or in more extreme direction, to stable hypoxia [41]. The identified set therefore reflects genes that are regulated differently by oscillatory hypoxia compared to stable hypoxia (Osc genes).

**Figure 4:**
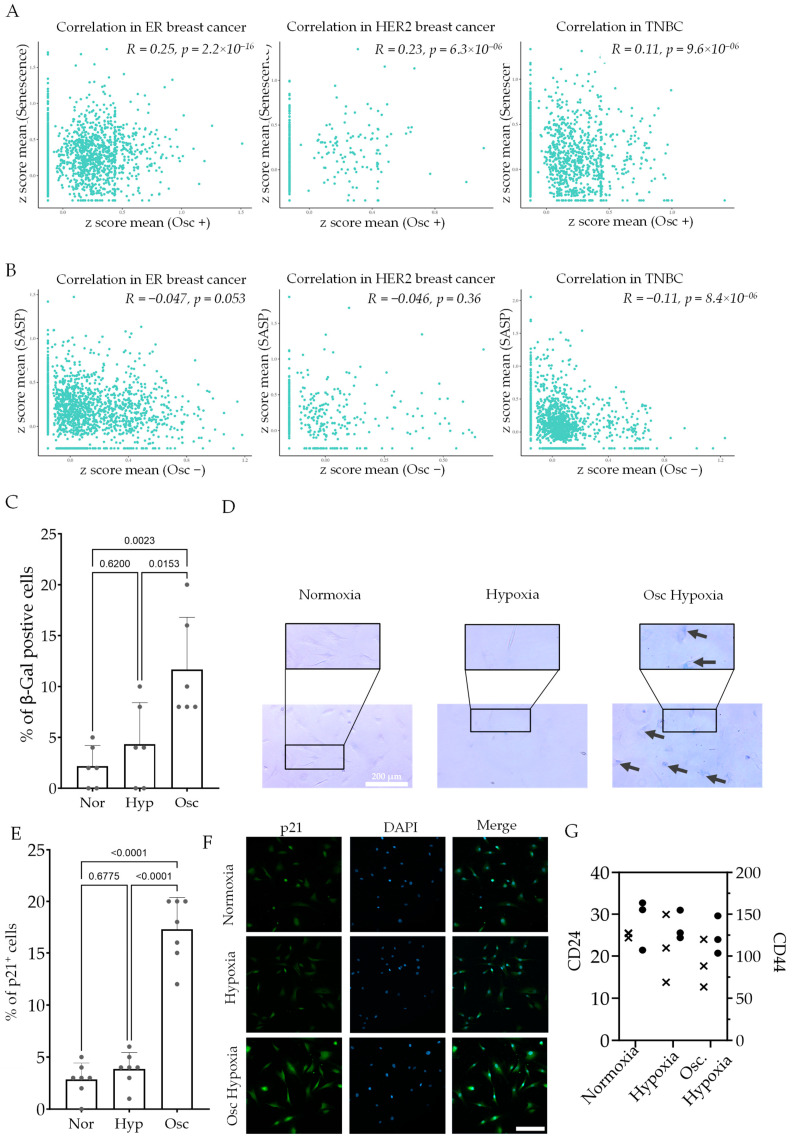
Oscillatory hypoxia induces senescence in ASCs. (**A**) Correlation in single-cell MSCs between the computed downstream gene expression-based z-score for senescence and genes upregulated in oscillatory hypoxia (Osc +) in breast cancer samples: ER+, HER2+, and TNBC patients. (**B**) Correlation in single-cell MSCs between the computed downstream gene expression based z-score for SASP (senescence-associated secreted proteins) and genes upregulated in oscillatory hypoxia (Osc +) in breast cancer samples: ER+, HER2+, and TNBC patients. (**C**) Percentage of ASC cells with β-Gal positive state in Normoxia, Hypoxia, and Oscillatory hypoxia with representative images shown in the (**D**). Black arrows show the β-Gal-positive cells in Oscillatory hypoxia. The scale bar is 200 microns. (**E**) Percentage of p21 positive cells after treating ASCs with Normoxia, Hypoxia, and Oscillatory hypoxia with representative images shown in (**F**). p21 (green), DAPI (blue); scale bar = 200 µm. (**G**) TPM (transcripts per million) values of CD24 (x), and CD44 (•) in MDA-MB-231 cells conditioned with Normoxia, Hypoxia, or Oscillatory hypoxia for >48 h.

Using published single-cell RNAseq data [42] from breast cancer patients, we separated mesenchymal stromal cells based on the published classification criteria. Although the genes regulated by oscillatory hypoxia (defined as Osc^+ve^: genes specifically increasing in expression in oscillatory hypoxia, and Osc^−ve^: genes specifically decreasing in expression in oscillatory hypoxia) may have cell context dependence, we asked if these genes correlate within stromal cells with any key phenotype. We created a single cell-specific score for gene set activation for Osc genes and compared it to other phenotypically meaningful gene sets. We surprisingly found that Osc^+ve^ genes showed a significant correlation with the senescent signature, particularly for ER+ve and HER2+ breast cancer cells (Figure 4A). Furthermore, although the effect size was small, we found a significant negative correlation for TNBC breast cancer cells between Osc^−ve^ and SASP (senescence-associated secretory phenotype) signatures (Figure 4B). Although it is not possible to ascertain whether MSCs with Osc signatures indeed were in unstable hypoxia locality, the correlation between the Osc gene signature and cellular senescence is indicative. We therefore sought to test the hypothesis by subjecting ASCs with stable or oscillatory hypoxia.

Molecular O_2_ has an extremely low solubility in aqueous solutions [43]. For long-term stable hypoxia treatment, a hypoxic ambient atmosphere created by a hypoxic chamber or incubator may suffice, but for fluctuating hypoxia, the change in bioavailable O_2_ is expected to lag behind ambient changes in O_2_ tension. We therefore cultured ASCs on a substrate permeable to O_2_ to allow cells to sense O_2_ directly from their adhered bottom side. Cells were cultured in stable 1% and fluctuating (cycles of 1% O_2_ for an hour and 21% for 30 min) O_2_ for 24 h, reflecting published physiological oscillations in O_2_ tension. The β-galactosidase assay showed that while stable hypoxia did not have a significant effect, oscillatory hypoxia resulted in a significant increase in the percentage of β-gal cells (Figure 4C,D). Similarly, immunofluorescence-based p21 labeling showed a significant increase in intensity only in oscillatory hypoxia (Figure 4E,F). To note, the mRNA levels of MDA-MB-231 cells did not show any significant change by hypoxia or oscillatory hypoxia for stem cell markers, CD24- and CD44-encoding genes (Figure 4G). These data confirmed that hypoxic oscillation could induce senescence in ASCs, which owing to the changed secretory phenotype could regulate the breast epithelial cell phenotype.

### 3.5. Senescent ASCs Enhance Migration and Stromal Invasion in MCF10A1 Breast Epithelial Cells

**Figure 5:**
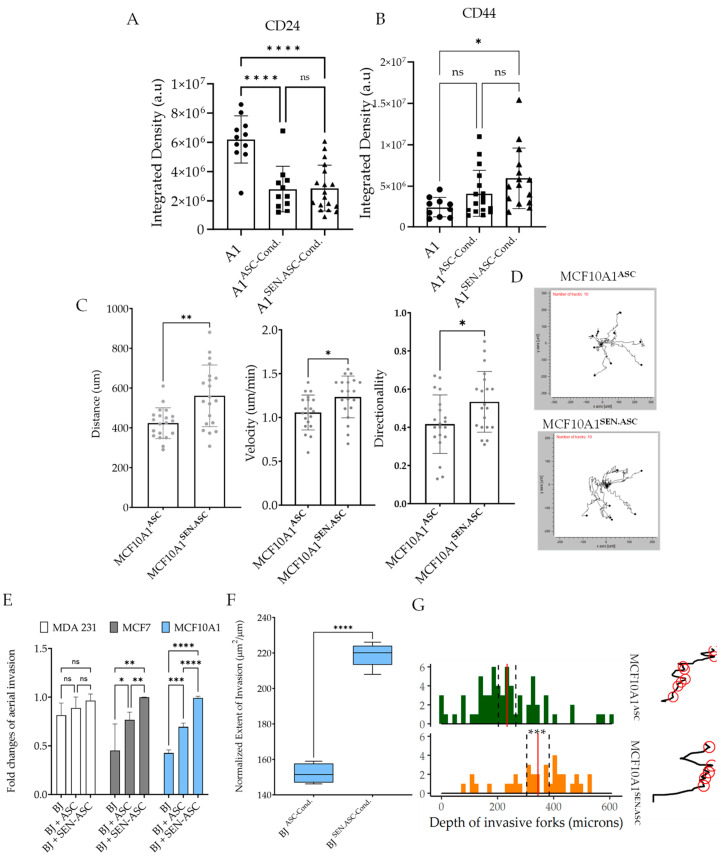
Senescent ASCs enhance MCF10A1 migration and invasiveness into stroma. (**A**,**B**) Immunofluorescence-based analysis of the total CD24 (**A**), and CD44 (**B**) levels in MCF10A1 cells treated with conditioned medium from ASCs or senescent ASCs. (**C**) Displacement, velocity, and directionality of MCF10A1 cells after conditioning with ASC and SEN-ASC media. (**D**) Live microscopy-derived trajectories of MCF10A1 cells’ migration with ASC and SEN-ASC media. (**E**) Fold changes for aerial invasion into the BJ stromal compartment for MCF7, MDA-MB-231, and MCF10A1 cells, without, or in co-culture with ASCs and SEN-ASC. (**F**) Normalized extent of the invasion of MCF10A1 cells into the BJ monolayer, conditioned with ASC media vs. SEN-ASC media. (**G**) Distribution of the depth of invasive forks (stromal dissemination by leader cells) of MCF10A1 cells conditioned with ASC media vs. SEN-ASC medium; representatives interface traces show tips of invading forks (red circle) at 24 h. In the above figures, statistical significance by t tests established at *p* < 0.05 and denoted by * *p* < 0.05, ** *p* < 0.01; *** *p* < 0.001, **** *p* < 0.0001.

As the differential gene expression signature for oscillatory hypoxia correlated with senescence and oscillatory hypoxia increased senescence in ASCs, we asked if paracrine interaction with senescent ASCs could further increase MCF10A1 invasion. As reprogramming to cancer stem cells’ state could potentially be indicative of the effect of senescent ASCs, we measured the levels of CD24, and CD44 after conditioning MCF10A1 cells with medium from ASCs and ASCs that were senesced (Figure 5A,B). ASCs could significantly decrease the CD24 levels, but increases in the CD44 levels were significantly achieved vs. the control only with senescent ASCs. These data suggest that senescent ASCs could enhance the cancer stem cells (CSC) phenotype in breast epithelial cells.

We then treated MCF10A1 cells with conditioned medium from ASCs and senescent ASCs for 24 h, and we measured their displacement over time in low confluence (Figure 5C,D). We found that while ASCs increased migration vs. the control, senescent ASCs further enhanced migration of MCF10A1 cells (Figure 5C,D). Indeed, both the velocity as well as the directionality of the cells were increased when conditioned with senescent ASC medium. We then used ANSIA to test if conditioning with senescent ASCs could further enhance the invasive capability of MCF10A1 cells than those treated with medium from wild-type ASCs. Here, 24 h of live microscopy-based observation showed that when ASCs were senesced, their capability to increase MCF10A1 invasiveness increased significantly more than the control ASCs, suggesting that senescent ASCs could contribute to breast cancer progression (Figure 5E,F). Morphological analysis of the invasive front showed that MCF10A1 cells treated with senescent MSCs showed many deep invasive forks formed in the stromal compartment (Figure 5G). Overall, these data show that senescent ASCs could transform normal breast epithelium even further, potentiating them toward an invasive, cancer-like state.

## 4. Discussion

Breast cancers cause the second highest death rate in women after lung cancer and are strongly associated with obesity, both in occurrence and malignancy. How obesity, and adipose tissue in general, affect breast cancer pathogenesis is not well understood. Breast stroma is highly adipose-rich, and breast cancers contain a high adipose content [44]. Obesity transforms adipose tissue by inducing metabolic dysregulation and inflammation. In addition, breast cancers also exhibit hypoxia, which results in stabilization of the master regulator of oxygen response in cells, HIF-1 [45]. Stabilized HIF-1 can cause recruitment of mesenchymal stem cells from the periphery, many of which are likely the adipose-derived mesenchymal stromal cells from either local adipose or distal adipose tissues [13]. The presence of ASCs has been linked to an adverse prognosis in breast cancers [9]. Although ASCs have been linked to increased malignancy in breast cancers, as well as other aspects of cancer pathologies, including drug resistance [9,10,46,47], it is not clear if ASCs could directly influence the transformation of breast epithelia.

Here, we used our stromal invasion assay, which uses nanopatterned tactic cues, to anisotropically orient actomyosin assembly in epithelial and stromal cells, increasing the sensitivity of the readout for stromal invasion by epithelial cells [5]. Using this platform, which we have termed the Accelerated Nanopatterned Stromal Invasion Assay (ANSIA), we discovered that ASCs could not only expectedly increase the invasive capacity of MDA-MB-231 and MCF7A cells derived from aggressive, and non-aggressive breast cancer adenocarcinomas, but also, surprisingly, of MCF10A1 cells derived from untransformed breast epithelium. We have shown that this ASC-driven effect on MCF10A1 is paracrine, as the conditioned medium from ASCs was sufficient to enhance MCF10A1 aggressiveness. RNAseq of MCF10A1 cells treated with conditioned medium from ASCs showed increased activation of pathways associated with cell migration and angiogenesis, while decreases in the activation of cortical actin cytoskeleton. Although we did not functionally explore the angiogenic effect of ASCs on MCF10A1 cells, many genes, including HIF1A, VEGFA, VEGFC, and ANGPT1, were increased. Another notable finding was that the expression of many tight-junction desmosomes claudins encoding genes was dramatically reduced by ASCs, suggesting a loss in epithelial integrity necessary for invasion. In parallel, CDH2, which encodes N-cadherin, was increased.

Although hypoxia-induced recruitment of mesenchymal stromal cells to breast cancer is well documented [13,48], newer imaging modalities have revealed that hypoxia is not stable in many cancers and shows fluctuating behavior [49,50,51]. In a recent study, we have shown that oscillatory hypoxia could dramatically change the gene expression pattern in cancer cells in an unexpected fashion, wherein oscillatory hypoxia resulted in a specific gene expression signature than stable hypoxia [22]. We surprisingly found that ASCs in oscillatory hypoxia showed senescent characteristics. Single-cell RNAseq data for MSCs in published studies of breast cancer also showed that genes correlated with oscillatory hypoxia in breast cancers were correlated with the SASP phenotype [52]. We, therefore, tested if senescent ASCs showed a different response to the MCF10A1 cell phenotype, finding that senescent ASCs could further enhance MCF10A1 invasive capabilities, as well as potentially the stem cell phenotype.

Our findings add to the increasing knowledge about the role of ASCs in breast cancer pathogenesis, suggesting that they may not only contribute to the metastatic transition of an already formed lesion but may also directly affect the cancerous transformation of normal breast epithelium. This finding is important because micrometastases of breast cancer before it is detected via non-invasive imaging modalities is now increasingly recognized [53,54]. If ASCs could directly increase breast epithelium invasiveness, they may contribute to micrometastases, and hybrid epithelial to mesenchymal transition [55,56,57]. Furthermore, in that hypoxia could increase ASC recruitment to the tumor site, unstable hypoxia could cause ASCs senescence, which could further enhance MCF10A1 invasiveness. Oscillatory fluctuations in hypoxia may occur in the tumor proper but may also result from obstructive sleep apnea (OSA), which has been linked to increased breast cancer occurrence [58,59]. Senescence of ASCs could also be an outcome of aging, inflammation, or metabolic syndrome, which could contribute to breast epithelium transformation, suggesting possible mechanisms of linkages between obesity and breast cancer occurrence [60,61]. The induction of senescence in ASCs under oscillatory hypoxia adds another layer to the complexity of stromal–epithelial interactions. Our data linking senescent ASCs to increased migration and invasion of breast epithelial cells suggest a potential pro-tumorigenic role for senescent stromal cells in breast tumorigenesis and the occurrence and onset of breast cancer.

## Data Availability

Publicly available datasets were analyzed in this study. This data can be found here: [PubMed PMID: 34493872 and PMID: 34062119]. Other data will be provided by email request to the Corresponding Author.
